# Non-parametric detection of atmospheric radon concentration anomalies related to earthquakes

**DOI:** 10.1038/s41598-018-31341-5

**Published:** 2018-08-29

**Authors:** Daichi Iwata, Hiroyuki Nagahama, Jun Muto, Yumi Yasuoka

**Affiliations:** 1 Department of Earth Science, Graduate School of Science, Tohoku University, Sendai, Japan; 2 Radioisotope Research Center, Department of Pharmacy, Kobe Pharmaceutical University, Kobe, Japan

## Abstract

Anomalous phenomena related to earthquakes have been studied to aid in the forecasting of large earthquakes. Radon (^222^Rn) concentration changes are known to be one of those phenomena. Many studies have quantified radon anomalies to identify physical aspects of radon emanations related to earthquakes. Here, we apply singular spectrum transformation, non-parametric analysis to estimate change points in time series, to atmospheric radon concentration. From 10 years of data from continuous observation of the atmospheric radon concentration over northeastern Japan and Hokkaido, we identify anomalies in the atmospheric radon concentration related to the moment releases of large earthquakes. Compared with a conventional model-based method, the singular spectrum transformation method identifies more anomalies. Moreover, we also find that change points in the atmospheric radon concentration prior to the 2011 Tohoku-Oki earthquake (*M*_w_ 9.0; 11 Mar. 2011, N38.1°, E142.9°) coincided with periods of other anomalous precursory phenomena. Our results indicate that singular spectrum transformation can be used to detect anomalies in atmospheric radon concentration related to the occurrences of large earthquakes.

## Introduction

Radon is a radioactive gas that belongs to the uranium series and has a half-life of approximately 3.8 days. Radon is released from the ground surface and measured as atmospheric radon concentration in radioisotope facilities^1^. In prior studies, radon concentration changes related to earthquake occurrences have been observed in air emanating from the soil^2^, in groundwater^3,4^ and in the atmosphere^1,5^. In the Kobe earthquake of 1995 (*M*_w_ 6.9; 17 Jan. 1995, N34.6°, E135.0°), the atmospheric radon concentration observed at the Kobe Pharmaceutical University increased starting two months before the main shock^1,6^. In recent research, anomalous atmospheric radon concentrations related to a shallow inland earthquake (*M*_j_ 5.5, depth 7 km; 5 Jul. 2011, N34.0°, E135.2°) in northern Wakayama was reported^5^. Several additional studies have reported observations of radon anomalies related to earthquake occurrences^7–10^, and some studies have experimentally verified mechanisms by which the concentration of radon can change when rock ruptures^11,12^. The long-term monitoring of atmospheric radon concentration and anomaly detection is expected to aid in earthquake forecasting.

Radon concentration in atmosphere is about one-hundredth and one-thousandth of concentration in soil air^13^ and in ground water^3^, respectively. Radon concentration in the atmosphere usually varies throughout the year because of seasonal factors^14^. Subsurface structural changes can involve exhalation of radon from the ground surface, and the atmospheric radon concentration reflects the averaged value of this exhalation over a wide area. Therefore, detecting anomalies in the atmospheric radon concentration related to earthquake occurrences is more difficult than those in soil gas radon or in ground water radon. In the previous studies, anomalies in the atmospheric radon concentrations were calculated by removing a seasonal factor estimated from a sinusoidal model and setting a threshold level for anomalies based on a normal variation period^15^. The results of these conventional methods depend on setting of the seasonal factor and normal variation period because the evaluation is determined based on deviations from the assumed physical model. A more quantitative understanding of the relationship between anomalies in the atmospheric radon concentration and earthquake occurrences requires an approach unencumbered by the ambiguities associated with model uncertainties.

Many data mining studies have been conducted to detect change points in a time series. With complex system data, constructing a model that describes the variation of observed values is difficult, as is detecting change points based on the model. Moreover, in some cases, the model-based method requires hypotheses on the stationarity of the data. In contrast, singular spectrum transformation (SST) was developed based on singular spectrum analysis^16^ to detect change points in complex time series^17^; SST requires neither generative models nor underlying physical models, and hence exhibits favorable performance with any data because of its pattern extraction capability. The pattern extraction is conducted via the following procedures: the time series is divided into subsequences, a matrix of the subsequence is constructed and a singular value decomposition is calculated on the matrix (see Methods and Supplementary Fig. S1). Because of this pattern extraction ability, SST is applied to analyze complex time series such as human behavior^18^ or meteorological observation data^19^. The SST result indicates a score of anomalousness that translates as change points in the time series. Therefore, the anomalousness of different time series can be compared quantitatively each other. In other words, using SST, we can detect anomalies in data and compare them each other without the ambiguities associated with model uncertainties. Therefore, SST is appropriate to detect anomalous changes in atmospheric radon concentration and to identify a relationship between atmospheric radon concentration and seismic activity.

**Figure 1:**
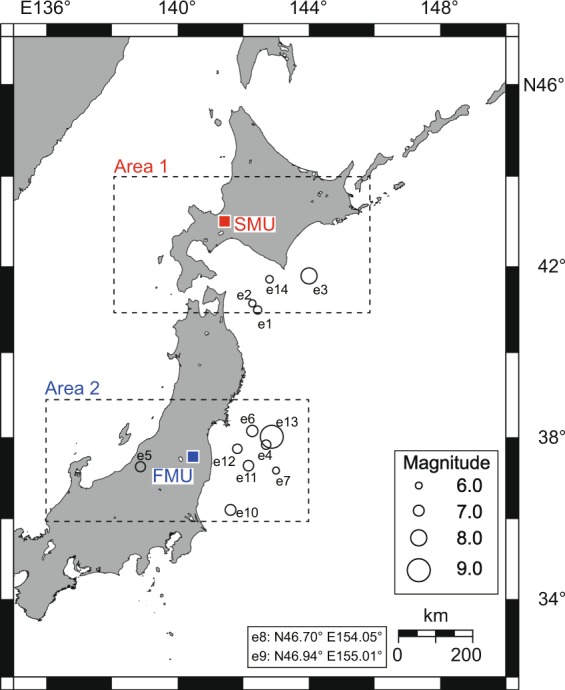
Map showing the location of atmospheric radon observation sites, Sapporo Medical University (SMU) and Fukushima Medical University (FMU). Black circles indicate the hypocenters of earthquakes, e1–e14 (e8: N46.7°, E154.1°, e9: N47.0°, E155.0°, details in Table 1). Dashed squares mark Area 1 (N41.0°–44.0°, E138.0°–146.0°) and Area 2 (N36.0°– 39.0°, E136.0°–144.0°), delineated to calculate cumulative seismic moment^39^ described in Fig. 2c,g. This map was generated using generic mapping tools (version 5.4.2) from an open source collection (http://www.soest.hawaii.edu/gmt/).

In the present study, the anomalousness of atmospheric radon concentration variation is calculated by SST using raw data (Supplementary Fig. S3) observed for approximately 10 years at Fukushima Medical University (FMU) (N37.7°, E140.5°) and Sapporo Medical University (SMU) (N43.1°, E141.3°)^20^ (Fig. 1). To show the effectiveness of SST in relation to the radon data, the SST result is compared with the result of the conventional method^15^. We then calculate the anomalousness of the cumulative seismic moment, and compare it with the anomalousness of the atmospheric radon concentration and evaluate the correlation between them quantitatively.

## Results and Discussion

**Figure 2:**
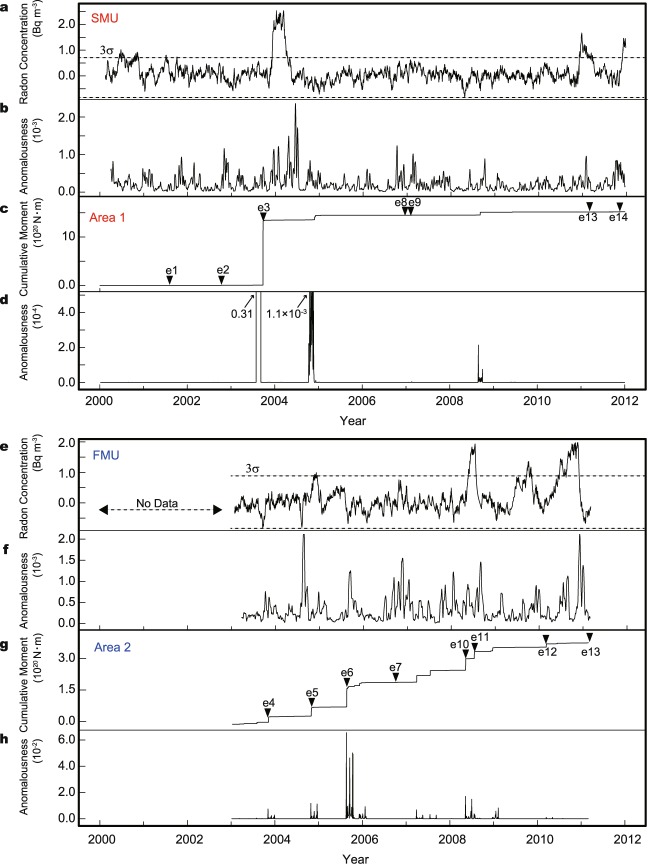
Results from the analysis of atmospheric radon concentration data observed at FMU and SMU. (**a**,**e**) Black solid curve indicates residual atmospheric radon concentration calculated by the conventional method; horizontal dotted lines indicate levels above and below three times the standard deviation (3σ) of atmospheric radon concentrations during normal variation periods (Data from the previous study^20^, **e** is modified from ref.^15^’s Fig. 7). (**b**,**f)** Anomalousness of radon concentration calculated by SST from raw data. (**c,g**) Black lines show cumulative seismic moment (*M* ≥ 3.0, depth ≥ 60 km^39^; inverted triangles indicate date of earthquake occurrences details in Table 1). (**d**,**h**) Anomalousness of cumulative seismic moment calculated by SST.

Anomalousness calculated by SST shows high level around the occurrences of larger earthquakes with magnitudes greater than 6.0 (events e3, e13, e14 in Fig. 2c and events e10–e13 in Fig. 2g; *M*_w_: moment magnitude, *M*_j_: the local magnitude calculated by Japan Meteorological Agency). Those high levels of anomalousness are considered to be change points of variation in the atmospheric radon concentration. These anomalies are also suggested by the conventional method which indicates high atmospheric radon concentration over the 3σ.

In addition, compared with the conventional method, SST also detects high levels of anomaly in the periods when atmospheric radon concentration did not exceed 3σ (Fig. 2a,e) but relatively larger earthquakes occurred (e4–e7 in Fig. 2g and e1, e2, e8, e9 in Fig. 2c). This indicates that the 3σ criteria in the conventional method is not enough to identify anomalies from data whose state drastically changes over time such as time series related to earthquakes, because detection capability of the conventional method depends on setting of normal variation period. The earthquakes which occurred around the periods of the high anomalousness of atmospheric radon concentration calculated by SST are plotted about epicentral distances and magnitudes in Supplementary Fig. S8. The previous study^21,22^ proposed that radon gas has sensitivity to earthquakes which causes crustal strain over 10^−8^. The earthquakes with atmospheric radon anomalies are plotted in the area with strain larger than the order of 10^−8^ (Supplementary Fig. S8). The anomalousness was also high from 2006 to 2007 (Fig. 2b) when there is no earthquake with magnitude larger than 6.0 in studied area (Area 1). However, two large earthquakes, e8 and e9, occurred east off the Kuril Islands (*M*_w_ 8.3; 15 Nov. 2006, N46.7°, E154.1° and *M*_w_ 8.1; 13 Jan. 2007, N46.9°, E155.0°). The crustal strain related to the earthquakes e8 and e9 is over 10^−8^ (Table 1 and Fig. S8). Consequently, in these periods, the earthquakes (Table 1) change the amount of radon exhalation indicating the high anomalousness calculated by SST. Moreover, anomaly in atmospheric radon concentration observed at SMU and FMU is suggested to have sensitivity to same one reported in previous study. Geodetic observations reported the post-seismic crustal deformation after events of e3 (Tokachi-Oki earthquake, *M*_w_ 8.0; 26 Sep. 2003, N41.8°, E144.0°)^23^ and around e7 (ref.^24^). After those earthquakes occurrences of large earthquakes were considered to be suppressed by releasing energy continuously by aseismic slip than around e5, e10 and e11 (Details in Supplementary Fig. S9)^25^.

**Table 1:** Large earthquakes that occurred when anomalousness calculated by SST was higher than anomalous level.

Event	Occurrence date	Location	Depth (km)	Magnitude (*M*_*j*_)	Hypocentral distance (km)
e1	August 14 2001	N41.00° E142.44°	38	6.4	246 (SMU)
e2	October 14 2002	N41.15° E142.28°	53	6.1	225 (SMU)
e3	September 26 2003*	N41.78° E144.01°	45	8.0 (*M*_w_)	262 (SMU)
e4	October 31 2003	N37.82° E142.70°	33	6.7 (*M*_w_)	196 (FMU)
e5	October 23 2004	N37.29° E138.87°	13	6.8	148 (FMU)
e6	August 16 2005	N38.15° E142.28°	42	7.1 (*M*_w_)	167 (FMU)
e7	October 11 2006	N37.19° E143.00°	50	6.0	230 (FMU)
e8	November 15 2006	N46.70° E154.05°	30	8.3 (*M*_w_)	1081 (SMU)
e9	January 13 2007	N46.94° E155.01°	30	8.1 (*M*_w_)	1158 (SMU)
e10	May 8 2008	N36.23° E141.61°	51	7.0	192 (FMU)
e11	July 19 2008	N37.31° E142.16°	32	6.9 (*M*_w_)	155 (FMU)
e12	March 14 2010	N37.72° E141.82°	40	6.7 (*M*_w_)	119 (FMU)
e13	March 11 2011**	N38.10° E142.86°	24	9.0 (*M*_w_)	214 (FMU) 576 (SMU)
e14	November 24 2011	N41.70° E142.80°	43	6.2	196 (SMU)

Event labels those shown in Fig. 2c,g. This list is compiled from data provided by the Japan Meteorological Agency^39^. *2003 Tokachi-Oki earthquake, **2011 Tohoku-Oki earthquake.

**Figure 3:**
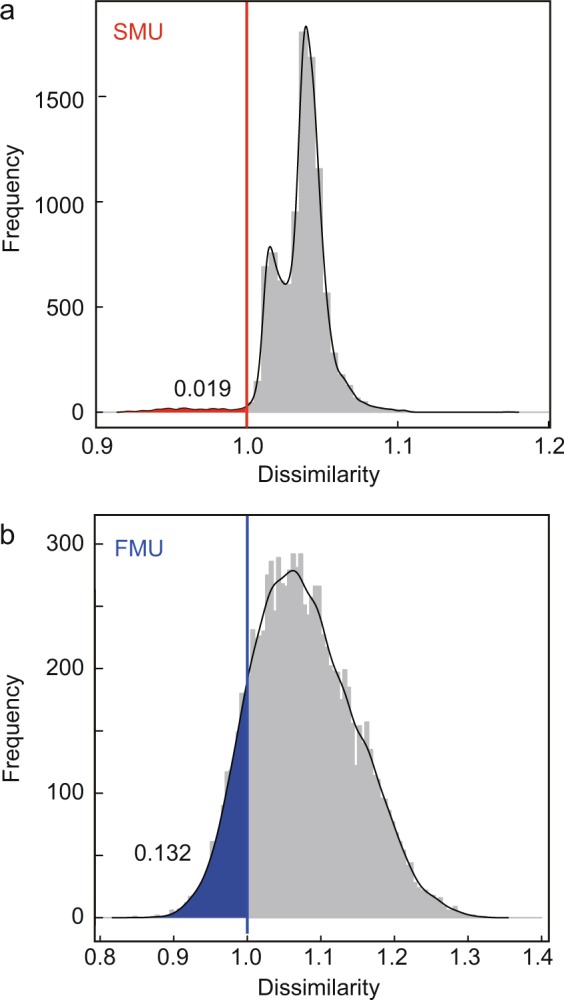
Comparison of anomalousness of radon concentration and anomalousness of cumulative seismic moment for random samples. (**a**,**b**) Histograms show distribution of dissimilarity between anomalousness of radon concentration and random samples. Vertical lines show dissimilarities between anomalousness of radon concentration and cumulative seismic moment of earthquake.

Figure 2d,h show the anomalousness of cumulative seismic moment (Fig. 2c,g) near the radon observation site (Area 1 and Area 2 in Fig. 1). To evaluate whether or not there is a correlation between the anomalousness of the radon concentration and that of the cumulative seismic moment, we calculate the anomalousness of randomly sampled time series that follow the seismic activity probability density, and compare them with the anomalousness of radon concentration (as detailed in Methods and Supplementary Fig. S2). The anomalousness of atmospheric radon concentration correlates with that of the actual cumulative seismic moment rather than that of random shuffled cumulative seismic moment (Fig. 3).

**Figure 4:**
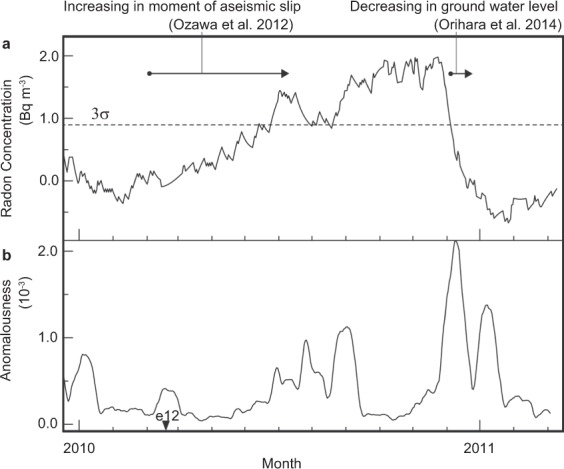
Time series of analyzed data from prior to the 2011 Tohoku-Oki earthquake (Jan. 2010–Mar. 2011) observed at FMU. (**a**) Residual atmospheric radon concentration calculated by conventional method. (**b**) Anomalousness of atmospheric radon concentration variation calculated by SST. Horizontal dotted lines in (**a**) indicate levels above and below three times the standard deviation (3σ) of atmospheric radon concentrations during normal variation periods.

Figure 4 shows the time series of analyzed data observed at FMU prior to the 2011 Tohoku-Oki earthquake (Jan. 2010–Mar. 2011). Atmospheric radon concentration had been increasing after a large earthquake on 14 March 2010 (*M*_w_ 6.7, e12 in Fig. 4b) and exceeded 3σ toward July 2010. During this period, the rate of increase was initially high, until July 2010; then, the concentration showed a high-level plateau toward the end of 2010. Subsequently, the atmospheric radon concentration decreased rapidly within two months (from November to December 2010, Fig. 4a). During this period, the anomalousness calculated by SST shows a rapid increase at the moment of the rapid decrease in the radon concentration, and then the anomalousness calculated by SST decreases (Fig. 4b). In spite of the variation in radon concentration, no large earthquake was reported until the Tohoku-Oki earthquake.

Before the earthquake, geodetic observations clarified crustal deformation by aseismic slip at the offshore of Fukushima^26^. The period of increase in atmospheric radon concentration coincides with the period of rapid increase in the moment of aseismic slip (Fig. 4a). An anomalous increase in the atmospheric radon concentration approximately three months before the main shock was also observed at SMU, which is 500 km from the epicenter (Fig. 2a). Radon concentration in the groundwater observed at Izu Peninsula, located 500 km from the epicenter, also increased three months before the Tohoku-Oki earthquake^27^. A concomitant decrease in the ground water level and water temperature in December 2010 was reported from a well in Iwate Prefecture (N39.2°, E141.7°, approximately 200 km from FMU)^28^. This period coincides with the period of rapid decrease in the atmospheric radon concentration (Fig. 4a), where SST indicates a high anomalousness value (Fig. 4b). Concomitant variations between the radon concentration and crustal deformation might also be related to aseismic slip events prior to the main shock.

Radon emanation from the ground surface depends on the ascending velocity of fluid and bubbles containing radon gas in cracks^29–31^. The velocity of the fluid depends on the fluid pressure, and the velocity of bubbles depends on the width of the cracks. In the present study, anomalies of atmospheric radon concentration detected by SST are found to correlate with the anomalies of the cumulative seismic moment (Figs 2 and 3). Seismic moment release associated with damage evolution causes a change in fluid pressure, which is proposed to cause radon anomalies^29^. In addition to this correlation, anomalies of atmospheric radon concentration also correlate with the periods of moment release caused by aseismic slip (Fig. 4). The aseismic slip around northeastern Japan causes the volumetric expansion of the area including the radon observation site^26^. Hence, the width of cracks in crustal rocks near the radon observation site increase with the aseismic slip, and the ascending velocity of bubbles containing radon gas increases as the crack widths increase^31^. Therefore, anomalies in atmospheric radon related to the aseismic crustal deformation are caused by the increase in the velocity of bubbles in cracks, in addition to changes in fluid pressure.

In the present study, we found that anomalies of atmospheric radon concentration could be associated with large earthquakes by SST. However, some of these anomalies were not detected by the conventional method. The objective and quantitative identification of anomalies by SST has the potential to enable the extraction of information for the pertaining to earthquake occurrences from observed data of precursory phenomena. Pre-seismic crustal deformation that is likely related to the preparation or nucleation process of the 2011 Tohoku-Oki earthquake was reported from geodetic and seismic observations near FMU^26,32,33^. Some anomalies in atmospheric radon detected by SST can be correlated to these transient crustal deformations before the large earthquake (Fig. 4), in addition to the pre-seismic change in groundwater level^28^. Above mentioned, crustal strain and variation of groundwater level affects emanation of atmospheric radon, which has been pointed out from the viewpoint of the change of ground water level before the large earthquake causes slow slip involved volumetric strain change^28^. This physical implication supports anomalies in atmospheric radon concentration with seismic activities. As a future work, application of SST to data measured across the country could lead to real-time monitoring of anomalous atmospheric radon concentration due to crustal deformation to evaluate seismic risks.

## Methods

### Observations of atmospheric radon concentration

Atmospheric radon concentrations were measured to monitor radioisotope leakage at Fukushima Medical University (N37.7°, E140.5°; measurement period: 2003–11 March 2011) and Sapporo Medical University (N43.1°, E141.3°; measurement period: 2000–2011), as shown in Fig. 1 and Supplementary Table S1. Outdoor air enters the radioisotope facility (intake duct height, intake volume and institute volume are detailed in Supplementary Table S1). The air is passed through high efficiency particulate air filters before being exhausted, and the exhaust air is monitored with a gas flow ionization chamber (model, effective volume and sampling flow rate are shown in Supplementary Table S1). In contrast to grab sampling, this procedure provides continuous measurement^34^. The ionization current can be converted to atmospheric radon concentration by the conversion factor (presented in Supplementary Table S1)^34^. The observed raw data is shown in Supplementary Fig. S3. The minimum detectable activity *A* (Bq m^−3^) is determined by the following equation^35^:

1 $$A=(4.65\sqrt{\frac{BG}{{T}_{count}}}+\frac{2.71}{{T}_{count}})Cf.$$

Here, *BG* (fA) is the background observation value; *T*_*count*_ (s) is the counting time and *Cf* (Bq m^−3^ fA^−1^) is the conversion factor. In the present study, *BG* is 21 (fA), *T*_*count*_ is 3600 (s) and *Cf* is 1.8 (Bq m^−3^ fA^−1^)^34^. The minimum detectable activity is then 0.64 (Bq m^−3^). The observed data in the present study (Supplementary Fig. S3) is reliable. The uncertainty of observed data is determined as the square root of the value of *BG* divided by *T*_*count*_, 7.6 × 10^−2^ (fA).

### Conventional method for analyzing radon anomalies

Observed value at radioisotope center contains a seasonal component and a linear trend. The data vary seasonally with minimum levels in summer and maximum levels in winter. The data also show gradual decreases with time. The observed linear decline in the data with time is a consequence of the radioactive decay of the calibration source in the monitor^5^. To estimate anomalous variation of atmospheric radon concentration related to earthquake occurrence, it is necessary to remove those components. In the conventional method, changes in atmospheric radon concentration are calculated by the following steps. First, a normal variation period (Supplementary Table S1) is set. In this study, we set the normal variation period based on the previous study^20^. Second, a seasonal component and a linear trend over the normal variation period are estimated with the least-squares method. Third, the seasonal and the linear trend components are removed from data over the entire period. Finally, 3σ is determined based on data in the normal variation period. Atmospheric radon concentrations higher than that level are identified as anomalous concentration (Fig. 2a,e). Anomalous atmospheric radon concentrations identified by the conventional method indicate the deviation from the assumed model. Therefore, the result shows positive and negative values around zero.

### Singular spectrum transformation

We used singular spectrum transformation (SST) to calculate anomalousness from raw atmospheric radon concentration data. SST is a non-parametric method based on singular spectrum analysis^16^, developed by Ide and Inoue^17^. Anomalousness refers to the change point in the time series, and is calculated based on following procedure. First, a time series is given (Supplementary Fig. S1):

2 $$x(1),\,x(2),\,\cdots ,\,x(t),\,\cdots ,$$

where *t* is time. The subsequence vector ***s***(*t*) is defined as below:

3 $${\boldsymbol{s}}(t-K)={(x(t-K),\cdots ,x(t-1))}^{{\rm{T}}},$$

where *K* is window width (Supplementary Fig. S1).

Second, a matrix *H*_1_(*t*) consists of ***s***(*t*) by

4 $${H}_{1}(t)=[{\boldsymbol{s}}(t-K-({r}_{1}-1)),\cdots ,\,{\boldsymbol{s}}(t-K-1),\,{\boldsymbol{s}}(t-K)].$$

This *K* × *r*_1_ matrix is called a trajectory matrix at *t*. The trajectory matrix *H*_1_(*t*) contains various change patterns in the past, where time is *t* − *K* − (*r*_1_ − 1) to *t* − 1. Third, a matrix is constructed on part of the future time series; this matrix is called the test matrix at *t*:

5 $${H}_{2}(t)=[{\boldsymbol{s}}(t-K-({r}_{2}-1)+L),\cdots ,\,{\boldsymbol{s}}(t-K+L-1),\,{\boldsymbol{s}}(t-K+L)],$$

where *L* is the lag between the two matrices *H*_1_(*t*) and *H*_2_(*t*). The parameter *r*_1_ and *r*_2_ denote the embedding dimension^36^. Fourth, the matrices *H*_1_(*t*) and *H*_2_(*t*) are decomposed using the following singular value decomposition:

6 $${H}_{1}(t)=U{{\rm{\Gamma }}}_{1}^{\frac{1}{2}}{V}_{1}^{{\rm{T}}},$$

7 $${H}_{2}(t)=Q{{\rm{\Gamma }}}_{2}^{\frac{1}{2}}{V}_{2}^{{\rm{T}}}.$$

Finally, ${U}_{p}^{(t)}$ and ${Q}_{s}^{(t)}$ consist of *U* and *Q*, which contain singular left vectors as follows:

8 $${U}_{p}^{(t)}=[{{\boldsymbol{u}}}^{(t,1)},{{\boldsymbol{u}}}^{(t,2)},\cdots ,{{\boldsymbol{u}}}^{(t,p)}],$$

9 $${Q}_{s}^{(t)}=[{{\boldsymbol{q}}}^{(t,1)},{{\boldsymbol{q}}}^{(t,2)},\cdots ,{{\boldsymbol{q}}}^{(t,s)}].$$

These matrices, ${U}_{p}^{(t)}$ and ${Q}_{s}^{(t)}$ are called the principal subspaces, and they indicate representative patterns in the trajectory matrix and test matrix, respectively. The anomalousness *a*(*t*) of the time series at time *t* is then calculated by comparing these principle subspaces:

10 $$a(t)=1-{\Vert {U}_{p}^{(t){\rm{T}}}{Q}_{s}^{(t)}\Vert }_{2},$$

where ${\Vert \cdot \Vert }_{2}$ represents the matrix 2 norm. SST is a method calculating how similar the trajectory matrix and the test matrix are.

Supplementary Fig. S4 shows an example of SST analysis applied to test data for the purpose of demonstration. The SST parameters were set to *K* = 30, *r*_1_ = 30, *r*_2_ = 30, *p* = 1, *s* = 1 and *L* = 7. Test data were generated by the following function:

11 $$\{\begin{array}{c}\sin (2\pi t)+N(3,\,0.3)\\ \sin (10\pi t)+N(3,\,0.3)\end{array}\,\begin{array}{c}(1\le t\le 400,\,601\le t\le 1000),\\ \,(401\le t\le 600),\end{array}\,$$

where *N*(*v*_2_, *v*_2_) indicates a normal distribution with a mean of *v*_1_ and a standard deviation of *v*_2_.

The parameters *p* and *s* are determined by calculating the singular value ratio. Supplementary Fig. S5 shows the singular value ratio for the example data above. The ratio of the first singular values is high. This is a representative pattern for the example data, which was given in that dimension. Therefore, in this test case, *p* and *s* were set to 1. Supplementary Fig. S6a,b show singular value ratios for the atmospheric radon concentrations observed at SMU and FMU, respectively. In both cases, the ratio of the first singular values is high, and therefore we set parameters *p* and *s* at 1. The parameter *K* in Equation (3) determines the sensitivity of the time periods: small or large *K* will inhibit the detection of anomalies. We calculate the anomalousness with *K* from 10 to 50, and thus determine that the appropriate *K* is 20 (SMU) and 30 (FMU) which can calculate the correlation between anomalies in atmospheric radon and earthquake well (Supplementary Fig. S7). There are several reports on continuous evolution of crustal deformation in the NE Japan island arc towards the catastrophic 2011 Tohoku-oki earthquake, such as earthquake migration, foreshocks and pre-seismic slow slip^26,32,33^. Except the foreshocks^33^, those continuous evolutions towards the large earthquake occurrence have time periods longer than *K* = 30 (FMU), 20 (SMU) days used in the present study. Therefore, SST with *K* of 30 (FMU), 20 (SMU) is considered to be able to detect the continuous evolution of the crust towards large earthquakes. In this study, SST parameters were set to *K* = 20, *r*_1_ = 20, *r*_2_ = 20, *p* = 1, *s* = 1 and *L* = 7 (Fig. 2b), *K* = 30, *r*_1_ = 30, *r*_2_ = 30, *p* = 1, *s* = 1 and *L* = 7 (Fig. 2f) to analyze atmospheric radon concentration data, and *K* = 20, *r*_1_ = 20, *r*_2_ = 20, *p* = 5, *s* = 5 and *L* = 7 (Fig. 2d), *K* = 30, *r*_1_ = 30, *r*_2_ = 30, *p* = 5, *s* = 5 and *L* = 7 (Fig. 2h) to analyze cumulative seismic moment data. SST detects changes in the mathematically defined subspace of the observation value (scalar value) in time series which don’t deal with physical space information.

#### Comparing similarity of time series of anomalousness

Because anomalousness calculated by SST is represented by values between 0.0 and 1.0, the results can be compared with each other even when the original time series are quite different. To evaluate the anomalousness similarity, we create random shuffled time series that follow the same seismic activity probability density. If the relationship between the anomalousness of radon concentration and that of cumulative seismic moment is by chance, the anomalousness of radon concentration should also correlate with that of the random shuffled time series. In this study, 10,000 time series of cumulative seismic moment are generated from random shuffled sample seismic activity, and their anomalousness are calculated by SST. The similarities between the true seismic time series and the random shuffled time series in relation to the radon concentration time series are calculated (Supplementary Fig. S2). To compare the similarities, dynamic time warping distances (DTW) are used^37^. DTW is an indicator for evaluating similarity of time-series. The simplest comparison between two time series is to use the Euclidean distance, but it is not robust to the time lag of features, then the similarity is underestimated. On the other hand, DTW can evaluate robustly to the time lag of features of time series. There are some time lags between anomalies of atmospheric radon concentration and that of seismic activity. Moreover, this study aims to evaluate the correlation between those anomalies. Therefore, DTW distance is considered to be appropriate.

### Cumulative seismic moment

The cumulative seismic moment is calculated to evaluate whether the seismic activity coincides with the anomalies in atmospheric radon concentration. Therefore, studied areas include the characteristic seismic zone, such as the subduction zone in Tokachi-oki (around N41.5°, E143.0°) and the subduction zone in Fukushima-oki (around N38°, E143.0°). Hence, we set the areas, Area 1 (N41.0°–44.0°, E138.0°–146.0°) and Area 2 (N36.0°–39.0°, E136.0°–144.0°), and calculated cumulative seismic moment for an earthquake with a depth of less than 60 km and a magnitude of higher than 3.0. The seismic moment *Mo*_*i*_ of the *i*-th earthquake in catalog is calculated by

12 $$M{o}_{i}={10}^{1.5{M}_{i}+9.1},$$

where, *M*_*i*_ is magnitude of the *i*-th earthquake^38^. Then, cumulative seismic moment *Mo*_*cum*_ from the start of catalog to the *n*-th is written by

13 $$M{o}_{cum}=\sum _{i=1,\cdots ,n}M{o}_{i}.$$

## Electronic supplementary material

Supplementary Information
