# Supplementary material for: Non-parametric detection of atmospheric radon concentration anomalies related to earthquakes
Source: Sci Rep. 2018 Aug 29;8:13028. doi: 10.1038/s41598-018-31341-5 (PMC6115410; doi:10.1038/s41598-018-31341-5)
Supplement: Supplementary file 1 — Supplementary Information [file 41598_2018_31341_MOESM1_ESM.pdf]

Non-parametric detection of atmospheric radon concentration anomalies related to earthquakes

Daichi Iwata*[1], Hiroyuki Nagahama[1], Jun Muto[1] and Yumi Yasuoka[2]

1 Department of Earth Science, Graduate School of Science, Tohoku University, Japan.
2 Radioisotope Research Center, Department of Pharmacy, Kobe Pharmaceutical University, Japan.

Supplementary Table S1

Measurement condition at FMU and SMU[20].

Sampling site	FMU	SMU
Duct height (m)	7	61
Intake volume (m^3 h^{-1})	7,850	8,600
Institute volume (m^3)	1,616	542
Model	Aloka-IC DGM-101*	Aloka-IC DGM-101*
Effective volume (L)	14	14
Sampling flow rate (L min^{-1})	6.5	6.5
Conversion factor (Bq m^{-3}) fA^{-1}	1.8	1.8
Measurement period (Year)	2003-11 March 2011	2000-2011
Normal variation period (Year)	2003-2007	2005-2009

* Hitachi Ltd., Japan

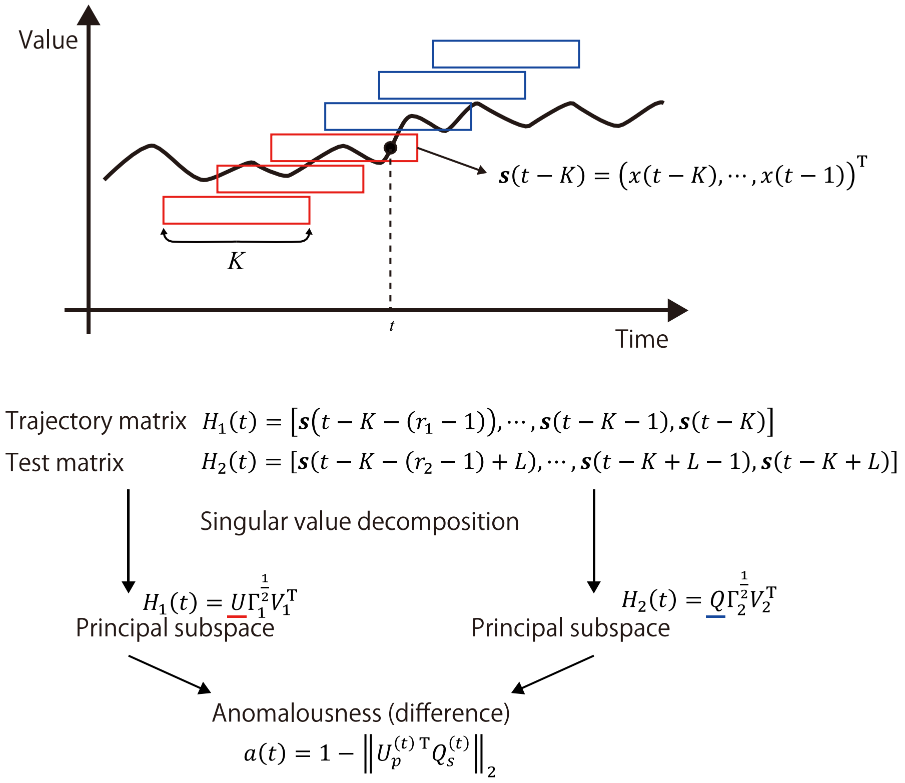

Supplementary Figure S1 Simplified schematic showing analysis of singular spectrum transformation.

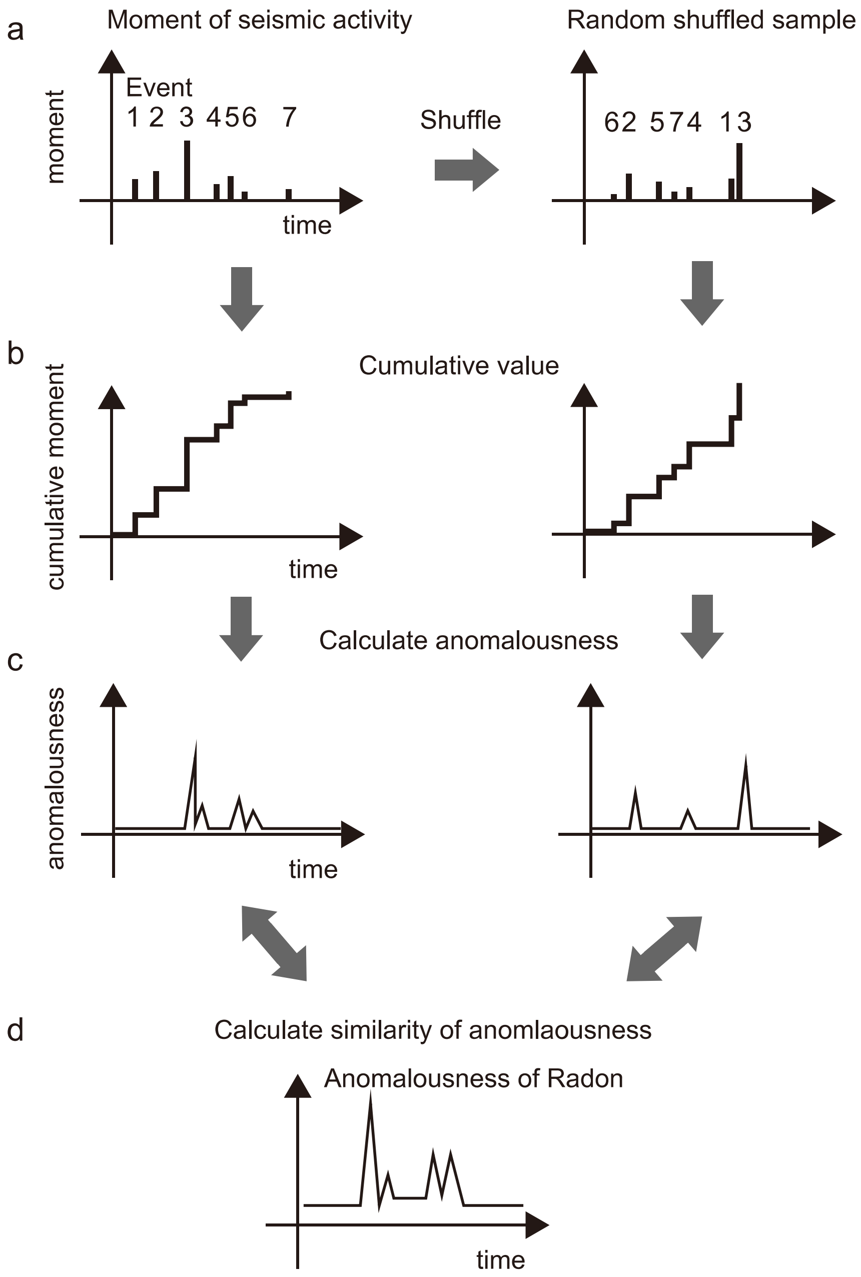

Supplementary Figure S2 Comparison of anomalousness of radon concentration and cumulative seismic moment for random samples. **a**, generate random shuffled samples. **b**, calculate cumulative time series. **c**, estimate anomalousness by SST. **d**, compare them with anomalousness of atmospheric radon concentration.

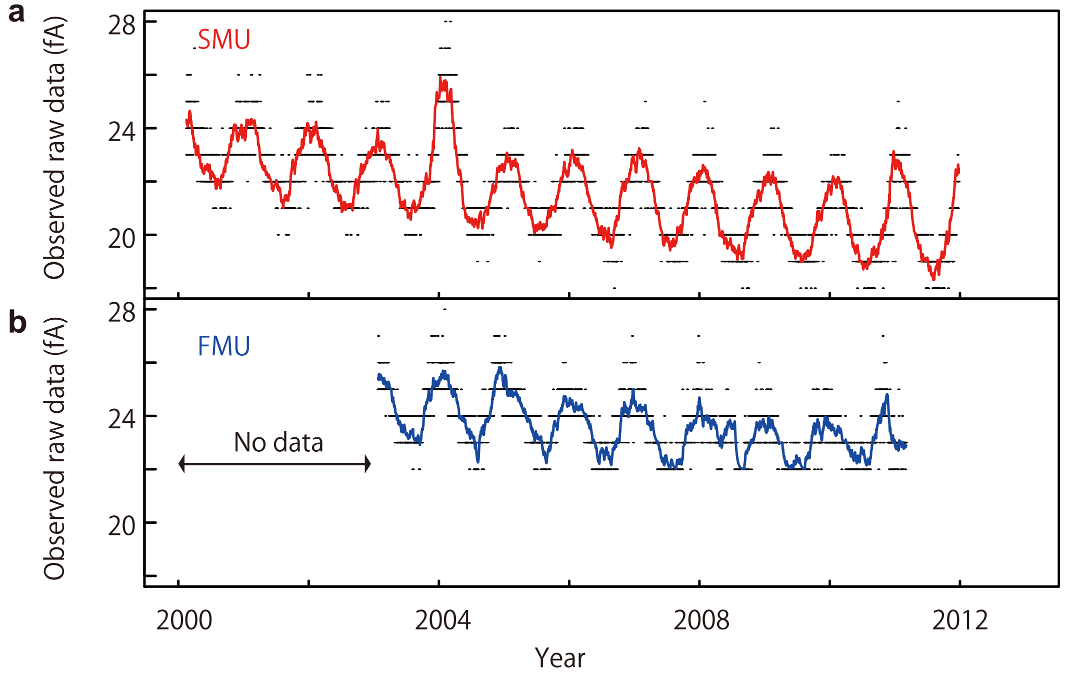

Supplementary Figure S3 The observation raw data of atmospheric radon concentration. Blue and red indicates raw data observed at FMU and SMU, respectively[20]. The variation of 1 (fA) is equal to the variation of about 1.8 (Bq m^{-3}).

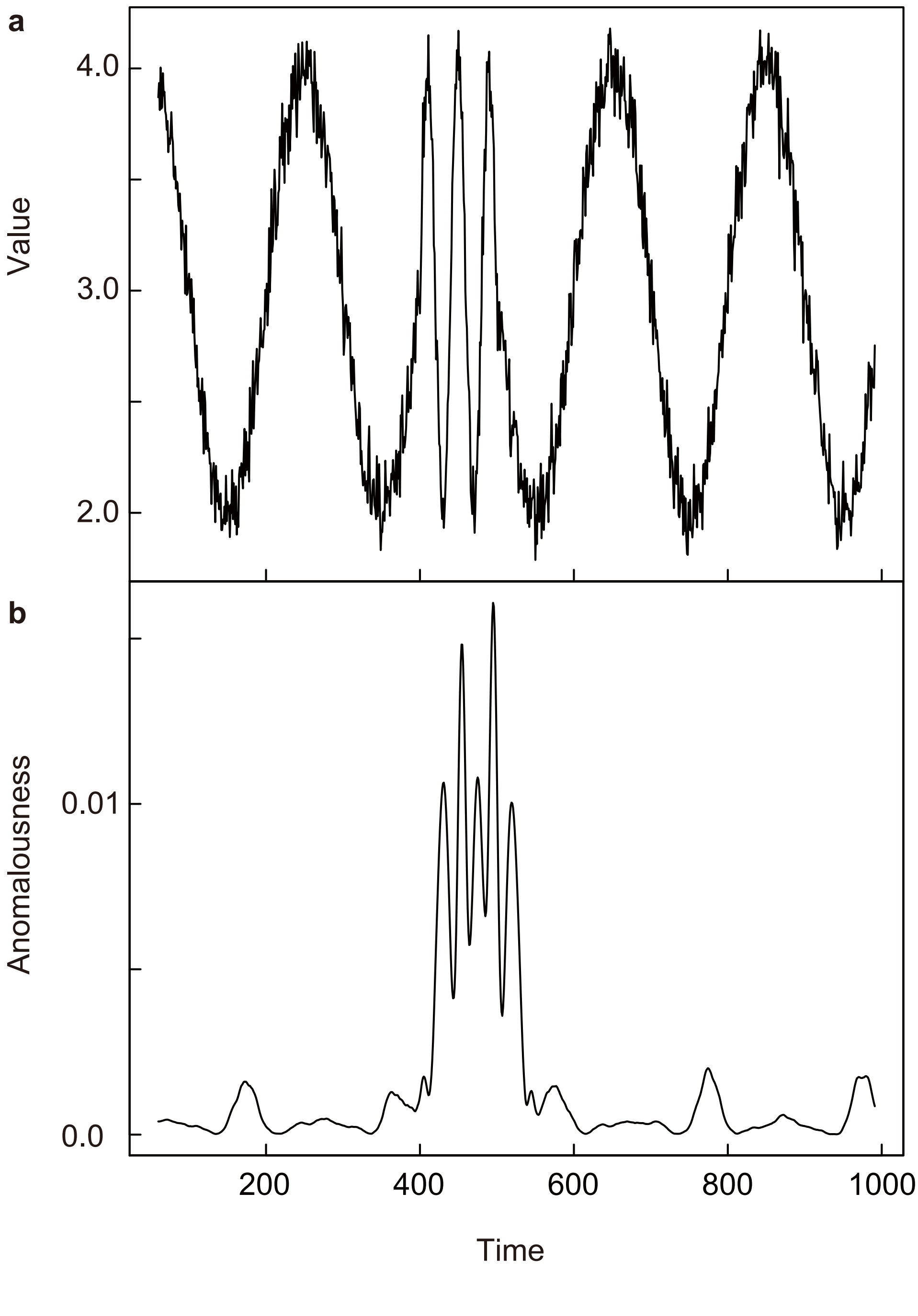

Supplementary Figure S4 Result for applying singular spectrum transformation (SST) to example data. a, Example data. **b**, Anomalousness calculated by SST.

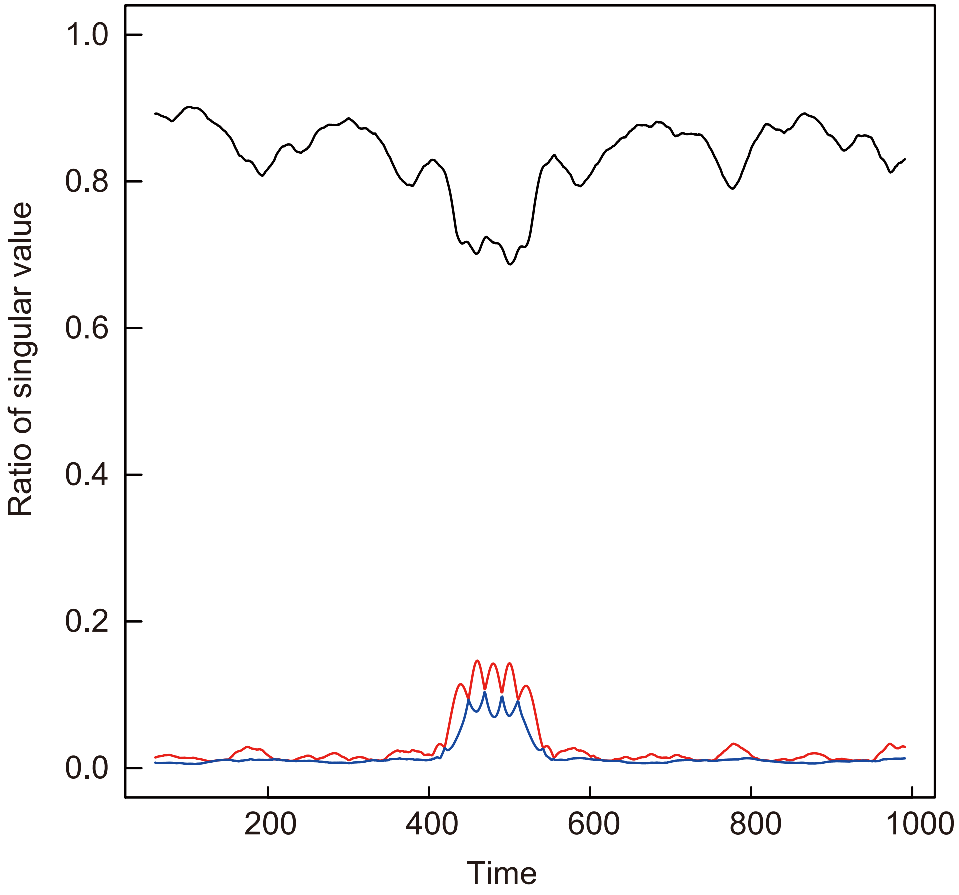

Supplementary Figure S5 Ratio of singular values about example data. Black, red, blue lines are ratio of first, second, third singular values, respectively.

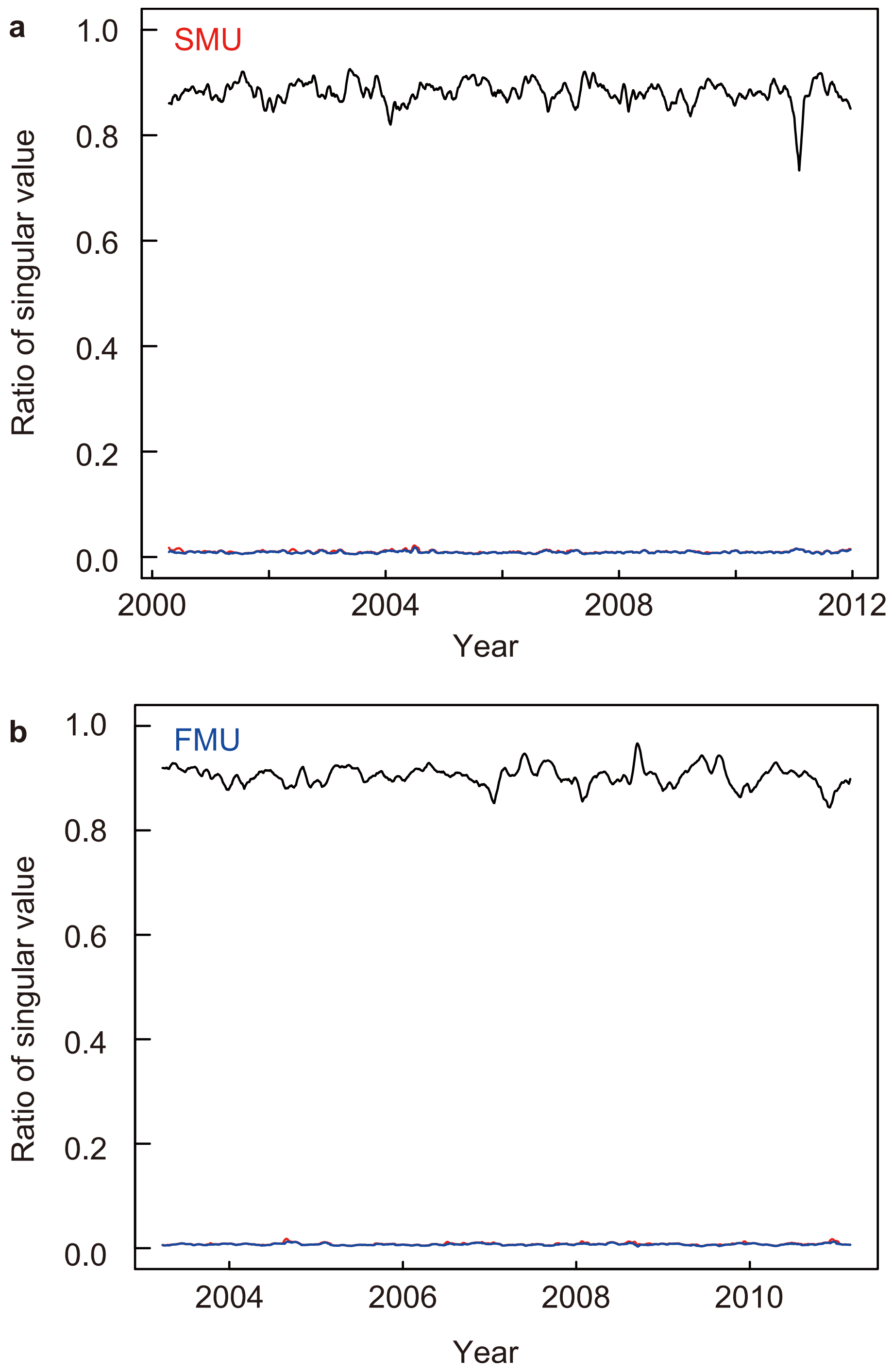

Supplementary Figure S6 Ration of singular values about atmospheric radon concentration. a, about data observed at FMU. **b**, about data observed at SMU. Black, red, blue lines are ratio of first, second, third singular values, respectively.

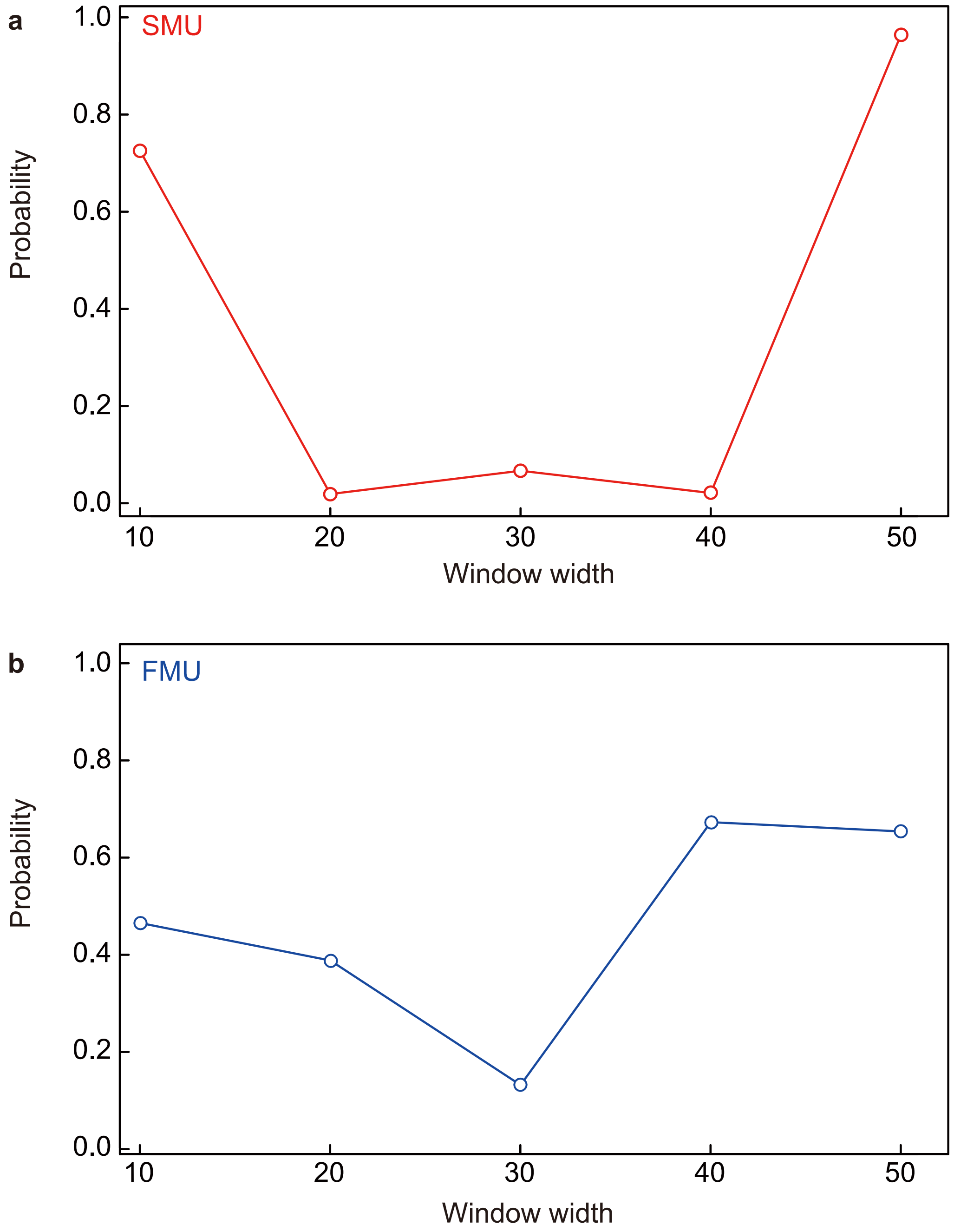

Supplementary Figure S7 Difference in analytical results using singular spectrum transformation with various windows width. The horizontal axis represents the window width of singular spectrum transformation. The vertical axis represents the probability of that the correlation between atmospheric radon concentration and seismic moment is coincidence by chance.

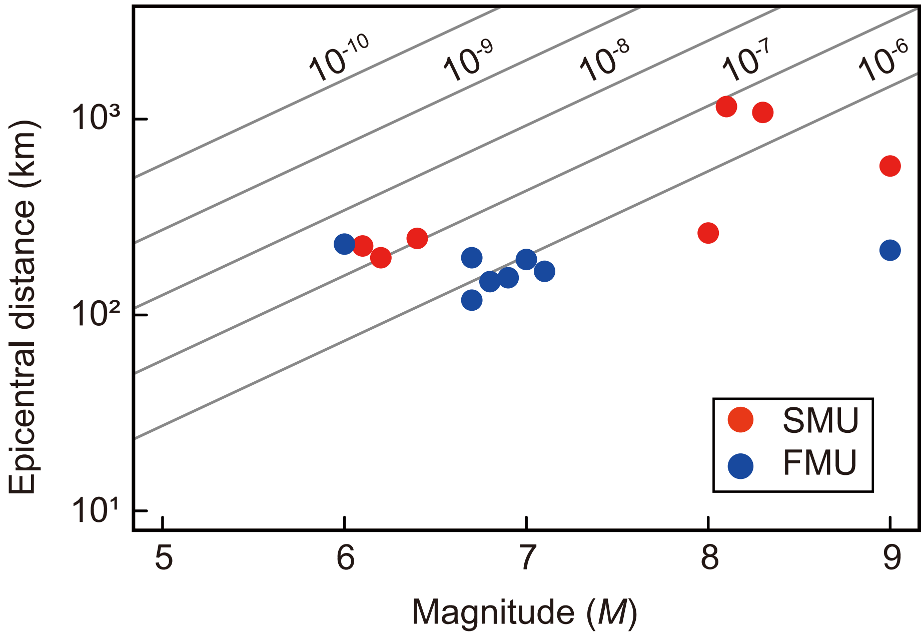

Supplementary Figure S8 Relationship of the magnitude and epicentral distance of anomalies by SST. Red and blue circles indicate earthquake which caused anomalies in atmospheric radon concentration (Red: SMU, Blue: FMU) detected by SST. Gray lines and values, 10^{-6} – 10^{-10}, show the crustal strain calculated with magnitude and epicentral distance of earthquakes (Table 1).

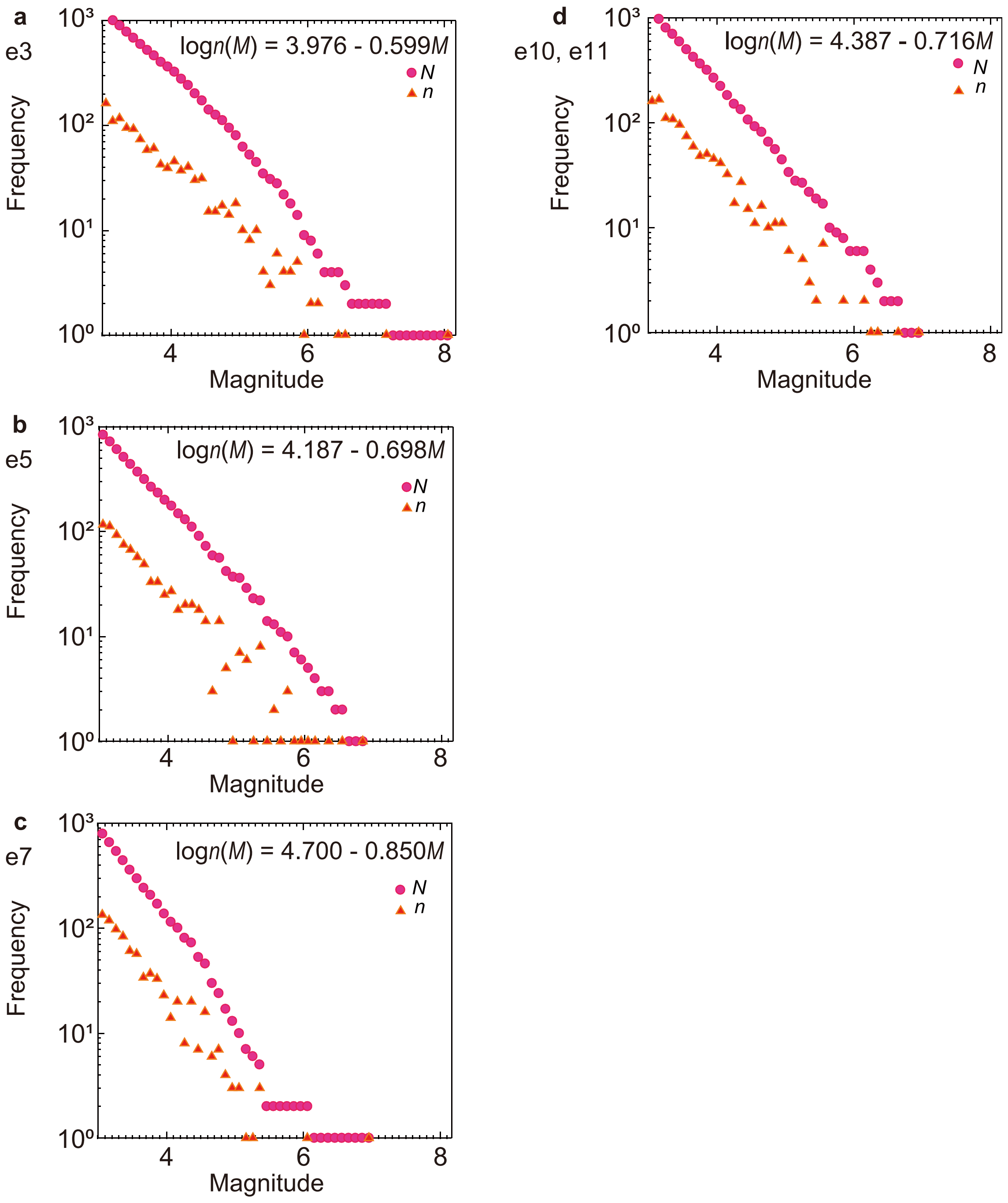

Supplementary Figure S9 The plots about magnitude versus the number of earthquakes. a shows the plot of Area1 (Fig.1) between Aug. 2003 and July 2004. **b-d** shows the plot of Area2 (Fig.1) between June 2004 and May 2005 (**b**), July 2006 and June 2007 (**c**), Jan. 2008 and Dec. 2008 (**d**). These periods include the earthquake occurrences e3, e5, e7 and e10-e11, respectively. In **a** - **d**, n and N indicate number of earthquakes and number of earthquakes having a magnitude $\geq M$, respectively. The plots are made from data provided by Japan Meteorological Agency[39].
